# 0092. Ischemic pre/post-conditioning protects the microcirculation in experimental sepsis

**DOI:** 10.1186/2197-425X-2-S1-P4

**Published:** 2014-09-26

**Authors:** D Orbegozo Cortes, S Fuhong, C Santacruz, K Hosokawa, K Donadello, J Creteur, D De Backer, J-L Vincent

**Affiliations:** Erasme Hospital, Dept of Intensive Care, Brussels, Belgium

## Introduction

Ischemic preconditioning induces complex physiological adaptations to improve the tolerance of cells and tissues to future ischemic episodes. This phenomenon has been most studied in myocardial cells but also occurs in other tissues.

## Objectives

Ischemic preconditioning could be used to improve the microcirculatory response to sepsis related hypoperfusion.

## Methods

Sixteen adult sheep (24-34 Kg) were anesthetized (midazolam, ketamine and morphine), mechanically ventilated and invasively monitored. Abdominal sepsis was induced by injecting autologous feces into the peritoneal cavity. Animals were randomly allocated to undergo remote ischemic preconditioning followed by intermittent post-conditioning (PRECON) or not (CONTROL). Controlled ischemic episodes of the lower extremities and pelvis were obtained by inflating an intravascular balloon in the aortic bifurcation. We performed 4 cycles of 2 min ischemia (4 min apart) 1 hour before sepsis induction and, thereafter, 1 inflation every 4 hours until death. Sublingual microcirculation was evaluated using side-stream dark field (SDF) video-microscopy capturing 5 videos of 12 sec at baseline and every 6 hours thereafter for later blinded analysis. We calculated the perfused vessel density (PVD), proportion of perfused vessels (PPV), mean flow index (MFI) and heterogeneity of PPV (PPV HI). Animals were followed until death or for a maximum of 30 hours. Data are presented as median values with inter-quartile ranges. Repeated measurement data were analyzed using a Generalized Estimating Equations approach in SPSS 19.0 (IBM,USA) with a p < 0.05 considered as significant.

## Results

See table 1.

**Table 1:** Table 1

		T0	T6	T12	T18	T24
**PVD (vessel/mm)**	**PRECON**	13.0 (12.8-15.8)	10.6 (9.8-12.1)	9.0 (8.6-11.1)	9.2 (8.5-10.1)	8.2 (7.3-9.3)*
	**CONTROL**	13.8 (12.5-15.7)	10.4 (9.5-11.3)	9.1 (8.6-10.8)	7.7 (6.9-9.6)	6.2 (5.0-6.7)
**PPV (%)**	**PRECON**	98 (97-99)	91 (89-93)*	86 (82-93)	84 (82-89)*	77 (76-83)*
	**CONTROL**	97 (97-98)	89 (86-90)	84 (82-87)	77 (66-84)	58 (50-65)
**MFI (0-3)**	**PRECON**	3.0 (2.9-3.0)	2.6 (2.5-2.7)	2.2 (2.1-2.6)	2.3 (2.1-2.6)*	2.2 (2.1-2.4)*
	**CONTROL**	2.9 (2.9-3.0)	2.4 (2.3-2.5)	2.2 (2.1-2.4)	1.7 (1.5-2.1)	1.2 (1.0-1.4)
**PPV HI (%)**	**PRECON**	3 (3-5)	9 (5-15)*	16 (11-40)	21 (18-39)	35 (25-39)*
	**CONTROL**	4 (2-5)	19 (13-25)	27 (23-35)	38 (21-55)	57 (44-66)

*[Sublingual microcirculatory variables]*

* = p < 0.05 compared with control group.

## Conclusions

Repeated ischemic pre- and post-conditioning of the pelvis and lower extremities protected the microcirculation in this sheep model of severe abdominal sepsis.

